# A Meteorological Table, by Dr. Higgins, of Brompton, from Jan. 22, to Feb. 21, 1805

**Published:** 1805-05-01

**Authors:** 


					A Meteorological Tabic, by Dr. Higgins, of Brompton,
from Jan. il'2, to Feb. '21, 1805.
i l'h> imomcter
Fahrenheit.
C;mc-
jrratic
U?
[y *?
1*3 C
P2
|3*
50
3J
28
28
28
31
33
34
*9
*3
29
35
44
19
34
44
5*
46
40
35
35
3?
35
33
3?
3*
3?
30
4*
34
33
31
35
29
30
29
36
35
33
30
32
39
42
46
33
39
49
54
54
40
39
39
40
38
40
37
37
31
3$
46
3^
29
28
29
28
28
30
3*
33
31
27
28
32
46
3*
28
38
48
48
44
34
33
29
34
34
18
30
30
28
3<>
48
0-55
below
0.55
1.66
below
1.66
1.II
1.66
2.22
1-66
O.55
below
1.11
0.00
3.88
5-55
7-7
?-55
388
9.44
12.22
12.
4.44
3.88
3-88
4.44
3-33
4.44
2.77
2.77
3-33
3-33
7-77
Uti^nt ot the iiuro-
meter. Inches.
ij u
5 c
US
U-g,
0 vz
29-33
.38
? j
?93
30.05
.16]
.18
29.41
lo|
41
129.28 29.27
'' ' .76
?49
?96
'0.08J
18
14
29.90
.29
.10
?57
o ?
8-
10
3?.
29
30
30.01
?3i
?54|
.18
29.03
30 07
?35|
29 92*29
30.08 30
.12
.01
?32
.70]
.60
?49,
?55
.42'
.29
.36!
?49
.25l
30.01
.10
I
? 20|
? II
29.68
II
19
Si
30.20
47
53
|z9.30|
?51
3?-35
29-93
.96
3C-I3
.01
?20;
.61
.65
.48
?55
?45
?34]
.29
?471
.38
.02
WEATHER.
Cloudy, fnow in the evening.
Snow.
Cloudy.
Cloudy*
Cloudy*
Cloudy.
Cloudy.
Cloudy, fnow in the everting;.
Cloudy, rain in the evening.
Cloudy.
Cloudy. . .
Fair in the morn, fnow in the evening*
Cloudy.
Cloudy, rain in the evening.
Showery.
Fair.
Cloudy, rain in the evening.
Showery.
Cloudy, rain in the night.
Cloudy.
Cloudy.
Fair.
Cloudy.
Fair.
Cloudy. ' , .
Fair.
Fair.
Fair.
Fair.
[Cloudy, rain in the evening.
;Rain.

				

